# The parental psychological distress caused by separation from their critically ill child during the COVID-19 pandemic: A tale of two cities

**DOI:** 10.3389/fped.2022.909210

**Published:** 2022-09-15

**Authors:** Anna Camporesi, Francisco Abecasis, Erica M. Torres, Elena Zoia, Francesca Izzo, Stefania Ferrario, Elisa Maria Teresa Melloni

**Affiliations:** ^1^Department of Pediatric Anesthesia and Intensive Care, Children’s Hospital “Vittore Buzzi”, Milan, Italy; ^2^Pediatric Intensive Care Unit, Centro Hospitalar Universitário Lisboa Norte, Lisbon, Portugal; ^3^Psychiatry & Clinical Psychobiology Unit, Division of Neuroscience, Scientific Institute Ospedale San Raffaele, Milan, Italy; ^4^University Vita-Salute San Raffaele, Milan, Italy

**Keywords:** PICU visitation policies, COVID-19, psychological distress, caregivers, separation

## Abstract

**Introduction:**

A child’s critical illness is a stressful event for the entire family, causing significant emotional distress among parents and changes to family functioning. The Severe Acute Respiratory Syndrome-Related Coronavirus 2 (SARS-CoV-2) pandemic has abruptly caused modifications in visitation policies of Pediatric Intensive Care Units (PICUs) in many countries. We hypothesized that caregivers with no or severely restricted access to PICUs would demonstrate increased psychological distress as compared to those who had limitless access (LA) to PICUs.

**Methods:**

Sociodemographic variables, levels of psychological distress, ratings of family functioning, and ability to cope with stressful events were collected with an online survey in a group of caregivers after their child’s hospitalization. Ratings of psychological distress were compared between caregivers with no/severely restricted (NA) and with LA to PICUs.

**Results:**

Measures of depression, anxiety, and global severity index (GSI) of psychological distress were significantly higher in NA caregivers as compared to LA. Among demographic characteristics of the sample, only gender influenced the severity of psychological symptoms: women showed an increased score on levels of somatization, depression, anxiety, and GSI. Avoidant coping style positively correlated with measures of depression. Univariate General Linear Model (GLM) analyses of the effects of sex, age, visitation policies of PICUs, and score of avoidant coping strategies on measures of psychological distress confirmed a significant univariate effect of no access to PICUs on parents’ psychopathological scores.

**Conclusion:**

Restrictions imposed on visitation policies in PICU during the pandemic negatively impacted families’ psychological wellbeing. A balance between the safety of patients, families, and health care professionals and meeting the needs of families is of utmost importance.

## Introduction

A child’s critical illness is a stressful event for the entire family, causing significant emotional distress among parents and changes to family functioning (1), which can lead to symptoms of Acute Stress Disorder (ASD), Post-Traumatic Stress (PTSS), and Post-Traumatic Stress Disorder (PTSD) (2–4).

Partnerships between families and the health care team are essential in pediatrics where children are often unable to self-report symptoms or treatment preferences due to their developmental stage or health status. Open visitation policies in Pediatric Intensive Care Units (PICUs) are the heart of Patient-Centered and Family-Centered care (PFCC), which is the scenario where there is a mutually beneficial partnership among patients, families, and providers, and the importance of the family in the patient’s life is recognized and valorized (5). The PFCC has been demonstrated to improve outcomes of patients, families, and health care providers. It can be effective in decreasing anxiety, sedative dose requirements, delirium, mechanical ventilation, and sedation duration, and it is significantly related to early mobility and reduced ICU length of stay (5, 6).

The pandemic of Severe Acute Respiratory Syndrome Corona Virus 2 (SARS-CoV-2) has abruptly caused modifications in visitation policies of PICUs across the globe (7), mainly necessitated by the scarcity of personal protective equipment (PPE) and the complexity involved in implementing protocols for the admission of family members.

Although it has been defined as ethical to limit visitation in the interests of public health during times of pandemic (8), the separation of a person from critically ill relative can be a source of stress (9, 10), which is particularly increased in the case of a hospitalized child with his parents (11).

The aim of the present study was to compare self-reported psychological distress between parents of children admitted to a PICU with no modification to visitation policy during the COVID-19 outbreak (and limitless parental presence) with parents of children admitted to a PICU with restricted visit access (from no presence allowed to access restricted to 1 h a day).

## Materials and methods

### Participants

The present is an observational, cross-sectional cohort study. After institutional review board (IRB) approval (2021/ST/005), the parents of children admitted to two PICUs for longer than 24 h during the period March–December 2020 were enrolled in the study. Parents of children whose outcome was death were not enrolled.

The two PICUs are located, respectively, in Milano, Italy (Vittore Buzzi Children’s Hospital) and Lisbon, Portugal (Centro Hospitalar Universitário Lisboa Norte).

The Italian PICU did not allow the entrance of families of admitted patients during the year 2020 up to October and allowed restricted access (1 h a day) during November and December 2020.

The Portuguese PICU did not change its visiting policy during the first year of the pandemic and allowed limitless presence at the bedside, as before the pandemic.

Parents of children who were admitted to both PICUs during the given period were contacted at the beginning of 2021 and agreed to participate in the study. The questionnaires were hosted on an online platform (*SurveyMonkey*^®^). Informed consent was obtained from all participants, and the IRB approved the study in accordance with the principles in the Declaration of Helsinki.

### Data collection and analyses

In the group of participants, sociodemographic data, levels of psychological distress, characteristics of family functioning, and coping skills were collected following their child’s hospitalization in the PICUs.

Patients’ clinical severity at admission to PICU was assessed with pediatric risk of mortality (PRISM) II (12).

Caregivers were asked to retrospectively evaluate their psychological distress using the self-report questionnaire Brief Symptom Inventory-18 (13) (BSI-18), an 18-item questionnaire that assesses three psychological symptoms (somatization, anxiety, and depression) and provides a global index of distress (global severity index; GSI) based on the number and intensity of the symptoms endorsed by the respondent.

Caregivers’ family functioning was assessed with the Family Assessment Device (FAD), identifying six dimensions of family functioning (14): (1) problem solving, the family’s ability to resolve problems at a level that maintains effective family functioning; (2) communication, which is defined as the exchange of information among family members; (3) roles, which evaluate established patterns of behavior for handling a set of family functions that include provision of resources, providing nurturance and support, and supporting personal development; (4) affective responsiveness, which assesses the extent to which individual family members can experience appropriate effect over a range of stimuli; (5) affective involvement, which is concerned with the extent to which family members are interested in and place value on each other’s activities and concerns; and (6) behavior control, which assesses how a family expresses and maintains standards for the behavior of its members.

The Brief-Coping Orientation to Problems Experienced (COPE) inventory (15, 16)was administered to measure caregivers’ effective and ineffective ways to cope with stressful circumstances. This scale can identify three coping styles:

1. Problem-focused coping, which is characterized by active coping, informational support, planning, and positive reframing.
2. Emotion-focused coping, relying on venting, emotional support, humor, acceptance, self-blame, and religion, and
3. Avoidant coping that includes strategies, such as self-distraction, denial, substance use, and behavioral disengagement.

Student’s *t*-tests exploring the effects of sex and visitation policies of PICUs on parents’ symptoms severity were performed. Pearson’s correlation analysis was conducted to test the correlation between age, years of education, economic status, family functioning scores, personal ability to cope with stressful events, and psychopathology scores. To account for the multiple covarying variables, we also tested the effect of predictors on the current psychopathological status (self-report scores) by modeling the influences of the predictors on the outcomes in the context of the General Linear Model (GLM) and calculating the statistical significance of the effect of the single independent factors on the dependent variables by parametric estimates of predictor variables (least squares method). Analyses of univariate effects were performed by using a commercially available software package (StatSoft Statistica 12, Tulsa, OK, United States) and following standard computational procedures (17, 18).

## Results

In total, 78 families in Lisbon and 20 families in Milano were contacted. Forty-three parents (*N* = 43) were agreed to participate in the study: 19 caregivers from Italy had no or severely restricted access to PICUs (NA group), while 24 parents from Portugal had maintained limitless access (LA group). The study was carried out 2–9 months after the child’s hospitalization; this time frame was homogeneous in both groups.

Demographic and psychological characteristics of the whole sample and the two subsamples are resumed in Table 1.

**TABLE 1:** Demographic and psychological characteristics of the whole group and subgroups.

	All (*N* = 43)	NA (*N* = 19)	LA (*N* = 24)	χ/T	*p*
Sex (M/F)	9/34	1/18	8/16	5.04	0.02*
PRISM II	5.34 ± 4.28	3.60 ± 2.47	6.65 ± 4.91	–2.19	0.03*
BSI–Somatization	6.90 ± 5.32	8.31 ± 6.23	5.79 ± 4.28	1.57	0.12
BSI–Anxiety	14.27 ± 5.27	16.78 ± 4.76	12.29 ± 4.86	3.03	0.004*
BSI–Depression	12.39 ± 6.01	15.26 ± 5.63	10.12 ± 5.39	3.04	0.004*
BSI–Global severity index	33.58 ± 14.91	40.36 ± 15.03	28.20 ± 12.69	2.87	0.006*
FAD–Problem solving	1.94 ± 0.32	1.90 ± 0.35	1.97 ± 0.29	–0.70	0.48
FAD–Communication	2.22 ± 0.38	2.05 ± 0.38	2.35 ± 0.33	–2.55	0.01*
FAD–Roles	2.33 ± 0.38	2.28 ± 0.38	2.38 ± 0.39	–0.76	0.45
FAD–Affective responsiveness	2.12 ± 0.51	1.96 ± 0.40	2.25 ± 0.55	–1.8	0.07
FAD–Affective involvement	2.77 ± 0.33	2.78 ± 0.28	2.77 ± 0.38	0.04	0.96
FAD–Behavioral control	2 ± 0.35	2 ± 0.39	2 ± 0.32	0.08	0.93
FAD–Global functioning	1.89 ± 0.46	1.79 ± 0.36	1.98 ± 0.51	–1.25	0.21
Brief COPE–Problem-focused therapy	1.91 ± 0.34	1.93 ± 0.33	1.90 ± 0.36	0.29	0.77
Brief COPE–Emotion focused therapy	1.66 ± 0.42	1.61 ± 0.48	1.69 ± 0.37	–0.57	0.56
Brief COPE–Avoidant coping	0.82 ± 0.38	0.78 ± 0.33	0.85 ± 0.41	–0.52	0.60

Caregivers self-rated their symptoms on the Brief Symptom Inventory-18 (BSI-18), also yielding mean scores for somatization, anxiety and depression; their family functioning at Family Assessment Device (FAD) providing mean scores for the following measures: problem solving, communication, roles, affective responsiveness, affective involvement, behavior control, global functioning and their ability to cope for stress with The Brief-COPE (Coping Orientation to Problems Experienced) inventory. Data are means ± standard deviations and χ squared, *T*-test statistics and relative *p*-values. Significant differences are marked with an asterisk (*).

In total, 49% of caregivers were aged between 26 and 40 years old and 51% were between 41 and 60: 53% of them were married, 17% were separated or divorced, and 30% were single. Concerning their educational level, 5% of parents reported to have a primary school diploma, 32% of parents reported to have a secondary school diploma, 23% of parents reported to have a high school diploma, 28% of parents reported to have a Bachelor’s Degree, 7% of parents reported to have a Master’s Degree, and 5% of parents reported to have a first-level Specializing Master. A full-time job was reported by 70% of respondents, 21% had a part-time job, 5% were housewives, and 4% were unemployed. Regarding annual income, 28% of respondents had an average salary lower than 8,000€, 21% of respondents had an average salary between 8,000€ and 15,000€, 37% of respondents had an average salary between 15,000€ and 28,000€, 12% of respondents had an average salary between 28,000€ and 55,000€, and only 2% had higher than 75,000€.

Brief Symptom Inventory measures of depression, anxiety, and GSI of psychological distress were significantly higher in NA caregivers as compared to LA (*t* = 3.04, *p* = 0.004; *t* = 3.03, *p* = 0.004; and *t* = 2.87, *p* = 0.006, respectively).

Among demographic characteristics of the samples, only gender influenced the severity of psychological symptoms: women showed an increased score on levels of somatization, depression, anxiety, and GSI (*t* = 2.05, *p* = 0.04; *t* = 2.55, *p* = 0.01; *t* = 2.44, *p* = 0.01; and *t* = 2.66, *p* = 0.01, respectively). No significant effect of age, education, average annual income or marital status, or child’s clinical prognosis on measures of parents’ psychological distress was found.

Among dimensions of family functioning, affective responsiveness was found to inversely correlate with anxiety score (*R* = − 0.34, *p* = 0.03), but no other dimension was associated with symptomatology. Avoidant coping style positively correlated with measures of depression (*R* = 0.37, *p* = 0.02) and GSI (*R* = 0.35, *p* = 0.03).

Univariate GLM analyses of the effects of sex, visitation policies of PICUs, scores of affective responsiveness, and avoidant coping strategies on measures of psychological distress confirmed a significant univariate effect of visitation policies of PICUs (anxiety: β = 0.38, *F* = 5.83, *p* = 0.021; depression: β = 0.39, *F* = 6.75, *p* = 0.014; GSI: β = 0.37, *F* = 5.79, *p* = 0.022), avoidant coping style (somatization: β = 0.43, *F* = 5.96, *p* = 0.024; depression: β = 0.57, *F* = 14.48, *p* = 0.006; GSI: β = 0.55, *F* = 13.05, *p* = 0.01), but none of the other predictors (Tables 2, 3).

**TABLE 2:** Correlations between measures of family functioning at Family Assessment Device (FAD) and ability to cope with stress at the Brief-COPE (Coping Orientation to Problems Experienced) inventory and measures of psychological distress at the Brief Symptom Inventory-18 (BSI-18).

	FAD–Problem solving	FAD–Communication	FAD–Roles	FAD–Affective responsiveness	FAD–Affective involvement	FAD–Behavioral control	FAD – Global functioning	Brief COPE–Problem-focused therapy	Brief COPE–Emotion focused therapy	Brief COPE–Avoidant coping
BSI–Somatization	*R* = 0.08 *p* = 0.629	*R* = 0.098 *p* = 0.557	*R* = 0.087 *p* = 0.603	*R* = –0.085 *p* = 0.609	*R* = –0.151 *p* = 0.362	*R* = 0.254 *p* = 0.123	*R* = 0.157 *p* = 0.345	*R* = 0.131 *p* = 0.439	*R* = –0.037 *p* = 0.827	*R* = 0.35 *p* = 0.136
BSI–Anxiety	*R* = –0.008 *p* = 0.958	*R* = –0.199 *p* = 0.229	*R* = –0.083 *p* = 0.619	*R* = –0.341 *p* = 0.032*	*R* = –0.226 *p* = 0.171	*R* = 0.052 *p* = 0.756	*R* = –0.124 *p* = 0.456	*R* = 0.073 *p* = 0.667	*R* = 0.088 *p* = 0.603	*R* = 0.374 *p* = 0.021*
BSI–Depression	*R* = 0.02 *p* = 0.904	*R* = –0.205 *p* = 0.216	*R* = –0.096 *p* = 0.564	*R* = –0.307 *p* = 0.061	*R* = –0.101 *p* = 0.543	*R* = –0.017 *p* = 0.915	*R* = –0.165 *p* = 0.320	*R* = –0.076 *p* = 0.654	*R* = –0.091 *p* = 0.590	*R* = 0.284 *p* = 0.093
BSI–Global severity index	*R* = 0.032 *p* = 0.849	*R* = –0.118 *p* = 0.481	*R* = –0.036 *p* = 0.826	*R* = –0.225 *p* = 0.173	*R* = –0.179 *p* = 0.280	*R* = 0.103 *p* = 0.535	*R* = –0.053 *p* = 0.752	*R* = 0.047 *p* = 0.780	*R* = –0.009 *p* = 0.954	*R* = 0.354 *p* = 0.031*

Data are R Pearson correlation coefficients and relative *p*-values. Significant correlations are marked with an asterisk (*).

**TABLE 3:** Univariate GLM analyses of the effects of sex, visitation policies of PICU’s, scores of affective responsiveness, and avoidant coping strategies on measures of psychological distress.

	Sex	PICU’s policy	FAD–Affective responsiveness	Brief COPE–Avoidant coping
BSI–Somatization	*F* = 0.83 β = 0.159 *p* = 0.369	*F* = 0.845 β = 0.163 *p* = 0.364	*F* = 1.288 β = –0.213 *p* = 0.265	*F* = 5.967* β = 0.438 *p* = 0.02
BSI–Anxiety	*F* = 2.416 β = 0.236 *p* = 0.13	*F* = 5.832* β = 0.381 *p* = 0.021	*F* = 2.258 β = –0.441 *p* = 0.113	*F* = 2.241 β = 0.475 *p* = 0.423
BSI–Depression	*F* = 1.906 β = 0.202 *p* = 0.177	*F* = 6.758* β = 0.397 *p* = 0.014	*F* = 1.334 β = –0.363 *p* = 0.376	*F* = 14.485* β = 0.572 *p* = 0.006
BSI–Global severity index	*F* = 2.207 β = 0.22 *p* = 0.147	*F* = 7.712* β = 0.40 *p* = 0.009	*F* = 1.563 β = –0.37 *p* = 0.243	*F* = 13.051* β = 0.55 *p* = 0.001

Data are *F*-tests, beta coefficients, and relative *p*-values. Significant tests are marked with an asterisk (*).

## Discussion

To our knowledge, this is the first study directly comparing parental psychological distress between parents who have been denied access to the bedside during their child’s PICU stay and parents who did not experience separation from their child during PICU stay, during the COVID-19 pandemic period.

The results show a correlation between separation and parents’ psychological wellbeing: those who have been separated from their children during the pandemic show higher scores in anxiety, depression, and GSI, which is the sum of nine symptom dimensions: somatization, obsession-compulsion, interpersonal sensitivity, depression, anxiety, hostility, phobic anxiety, paranoid ideation, and psychoticism.

The experience of having a child admitted to a PICU is an extremely stressful one, with long-time repercussions, as easily understandable and objectively demonstrated by previous studies (4, 19, 20). During this experience, both the child (patient) and caregivers become vulnerable due to the uncertainties generated by the illness and the hospitalization. Coping with this new situation requires adaptation to it, and in this process, there is a potential role for health care workers to provide a more comfortable environment. The possibility for parents to be at the bedside without time restrictions had become obvious in a great part of the world before the COVID-19 pandemic that has abruptly changed this scenario (7). Its benefits—communication, collaboration, and support—show their effect on all the figures involved: the child who is critically ill, the family, and the health care workers. In 2020, a qualitative descriptive study was conducted at an Australian quaternary hospital to explore the care and communication experienced by family members of ICU patients during this time. The severe visiting restrictions introduced in the ICU during the pandemic to limit the spread of infection and protect patients and staff members have been reported to cause significant psychological and social impacts on families. Patient care and involvement in decision-making were appeared to be unchanged, but communication with staff was felt to be lacking (21).

The experience of not being allowed at the bedside, close to the child, adds on the opposite another stress to parents, as shown by our results. Similar results have been demonstrated by researchers in relatives of adult patients admitted with COVID-19 (9) but we believe family presence is inalienable and undeniable when the patient is a child, especially if the child is critically ill. In a potential and dramatic scenario, the patients could die without having their families with them, and a family could lose their child without being present. Although the initial intention is a good one (avoidance of community spread of pathogens), the results of a restricted visitation policy cause a too severe burden on the different actors involved that cannot be acceptable (22).

It is interesting to note that this psychological distress is not strictly related to the patient’s clinical prognosis/gravity (through the PRISM II index): Portuguese parents, though their children were on average more severely ill than the Italian ones, showed better results, as if the event of critical illness/PICU admission was the cause of stress *per se*, rather than the real clinical severity. In addition, this could be explained by better communication with nurses and doctors when the presence at the bedside of the child is guaranteed and a better understanding of the patient’s conditions and procedures a child is undergoing (23).

Among parents, women showed higher scores in some of the investigated points, namely, somatization, depression, and anxiety; although it is beyond our scope to interrogate the causes of this difference, this is a recognized scenario (24–26). However, our results suggested that a restricted visitation policy could impact the severity of anxiety, depression, and GSI scores regardless of the gender of the participants, which lacked significant effect in the models.

The severity of anxiety was found to inversely correlate with affective responsiveness in the family context. Anxiety is characterized by a condition of diffuse arousal following the perception of a real or imagined threat. This future-oriented, self-focusing emotion, when it reaches maladaptive levels, can consume a great number of attentional resources and lead to the feelings of helplessness and withdrawal. Therefore, it is no surprise that deficiencies in emotional awareness and affective responsiveness over a range of different stimuli are likely to associate with anxiety feelings.

The use of avoidant coping strategies to deal with stressful events did not differ in the two samples and was associated with a higher level of psychological distress.

Communication between health care workers and families has changed means, during the COVID pandemic, with the new strategies being implemented, such as video conferences, but the results in terms of satisfaction are never as satisfying as in person (27) and could lead to potential inequalities (28, 29) between families.

### Potential limitations

We acknowledge there are a few limitations to our study, mainly concerning the temporal distance of the Questionnaires’ administration to parents in relation to the PICU stay of their child. A previous study (19) evaluating the trajectories of parental distress up to 12 months after the experience of admission of a child to PICU showed three types of reactions—persistent low distress, persistent moderate-high distress, and high distress with recovery—but the pattern of distress did not rise over time. Based on this too, we believe that it is not likely that the participants in our study could be of over-reporting symptoms, although we cannot rule it out; the possibility, however, resides homogeneously in both groups along with the same time gap being present in both groups.

Another limitation that could be addressed is that the two study groups are from two different countries, but these two countries are similar in terms of geographical position, language, culture, and religion; and the feelings a parent experiences toward the child are universal.

### Strengths

The main strength of the study resides in the very well-detailed scales that were administered to the parents, which cover different aspects of the potential responses to psychological distress and of the different mechanisms of coping. Although we feared that the questionnaires could have been time consuming and tiring for “lay” persons, all parents were enthusiastic about their participation in the study and did not report fatigue in completing the questionnaires.

## Conclusion

Separation from a critically ill child during the most acute phase of the disease, that is, during PICU stay, has detrimental effects on parents in terms of psychological distress symptoms. The results of this study reinforce the need to develop strategies to allow the presence of parents during PICU admission, even in times of pandemic or other exceptional circumstances. The lessons learned from COVID-19 can be useful in future pandemics.

## Data availability statement

The raw data supporting the conclusions of this article will be made available by the authors, without undue reservation.

## Ethics statement

The studies involving human participants were reviewed and approved by Milano Area 1, protocol no. 2021/ST/005. The patients/participants provided their written informed consent to participate in this study.

## Author contributions

AC and EM: study design and conceptualization. FA, ET, FI, SF, and EZ: data collection. EM: data analysis. AC, EM, and FA: manuscript writing. All authors contributed to the article and approved the submitted version.
